# Designing Initiatives for Vulnerable Families: From Theory to Design in Sydney, Australia

**DOI:** 10.5334/ijic.3963

**Published:** 2019-07-25

**Authors:** John G. Eastwood, Denise E. De Souza, Miranda Shaw, Pankaj Garg, Susan Woolfenden, Ingrid Tyler, Lynn A. Kemp

**Affiliations:** 1 School of Women’s and Children’s Health, The University of New South Wales, Sydney, NSW, AU; 2 Ingham Institute of Applied Medical Research, Liverpool, NSW, AU; 3 Charles Perkins Centre, Menzies Centre for Health Policy, Discipline of Child and Adolescent Health, and School of Public Health, University of Sydney, Sydney, NSW, AU; 4 Sydney Institute for Women, Children and their Families, Sydney, NSW, AU; 5 Community Health Services, Sydney Local Health District, Level, Camperdown, NSW, AU; 6 Sydney Children’s Hospital Network, Sydney, AU; 7 School of Humanities, Nanyang Technological University, SG; 8 Dana Lana School of Public Health, University of Toronto, Toronto, ON, CA; 9 Fraser Health Authority, Surrey, BC, CA; 10 Translational Research and Social Innovation (TReSI), School of Nursing and Midwifery, Western Sydney University, Locked Bag, NSW, AU; 11 Discipline of Child and Adolescent Health, School of Medicine, University of Sydney, NSW, AU

**Keywords:** critical realism, evaluation, theory, developmental origins of health and disease, neighbourhood, social epidemiology, translational epidemiology, collaborative design, child, families

## Abstract

**Introduction:**

Intergenerational cycles of poverty, violence and crime, poor education and employment opportunities, psychopathology, and poor lifestyle and health behaviours require innovative models of health care delivery to break them. We describe a programme of research informed service development targeting vulnerable families in inner metropolitan Sydney, Australia that is designed to build and confirm a “Theory of Neighbourhood Context, Stress, Depression, and the Developmental Origins of Health and Disease (DOHaD)”. We describe the development of an intervention design and business case that drew on earlier realist causal and programme theoretical work.

**Methods:**

Realist causal and programme theory were used to inform the collaborative design of initiatives for vulnerable families. The collaborative design process included: identification of desirable and undesirable outcomes and contextual factors, consultation forums, interagency planning, and development of a service proposal.

**Results:**

The design elements included: perinatal coordination, sustained home visiting, integrated service model development, two place-based hubs, health promotion and strengthened research and analysis capability.

**Conclusions:**

We demonstrate here the design of interventions for vulnerable families in Sydney utilising translational research from previous realist causal and program theory building to operational service design. We have identified the importance of our earlier analysis of underlying causal mechanisms and related programme mechanisms for identifying the elements for the full intervention design. The application of theory added rigour to the design of the integrated care initiatives. In applying the theory to the local situation the analysis took into account: the role of the local agencies; evidence of program effectiveness; determinants and outcomes for local children and their families; the current deployment of service resources; and insights from front-line staff and interagency partners.

## Introduction

Inequities in the health and wellbeing of Australian children and families who live in disadvantaged communities are growing [1,2,3], despite a range of government initiatives designed to alleviate the impact of disadvantage and social exclusion [4].

Key to this is the issue of how we break the intergenerational cycles of poverty, violence and crime, poor education and employment opportunities, psychopathology, and poor lifestyle and health behaviours (including: unhealthy nutrition and physical activity, tobacco and substance use, interpersonal violence, early and unprotected sexual activity) [5]. Longitudinal cohort studies, some for three generations, have identified intergenerational transmission of psychopathology, poor parenting practices and family dysfunction that contribute poor health outcomes throughout the life course (including: suicide, teen age pregnancy, obesity, depression, tobacco smoking, diabetes and cardiovascular disease) [6]. Implicated as predictors of intergenerational transmission are: child abuse, harsh parenting practices and socio-economic disadvantage [7,8,9].

Increased understanding of the complex and inter-related issues that contribute to poor outcomes for vulnerable disadvantaged families have prompted concern from researchers and service providers about the often fragmented and inefficient service response, one that is not specific to local community needs [10]. This has prompted an increased policy commitment to community-led, multi-disciplinary, cross-sector integrated service delivery [11,12]. There is limited research on how to design or build an evidence-informed integrated response to complex social problems.

Critical realism offers an approach to empirically inform theory building and collaborative design of social interventions [13,14]. As a philosophy of science, it contends that there is a natural and social reality that exists independently of empirical observation and human thought. Those unobservable structures and mechanisms, under certain conditions, cause the observed events, and can be discovered and understood. Thus critical realism requires an understanding of the social situation or context, and requires the investigation of underlying mechanisms (causes) behind the observed events. In the study and practice of integrated care, the critical realist approach requires the inclusion of an analysis of pre-existing structures and mechanisms that may be contributing to the observed maternal, child and family outcomes [15], followed by an analysis of how an intervention may work to produce the desired change in observed outcomes.

**Figure 1:**
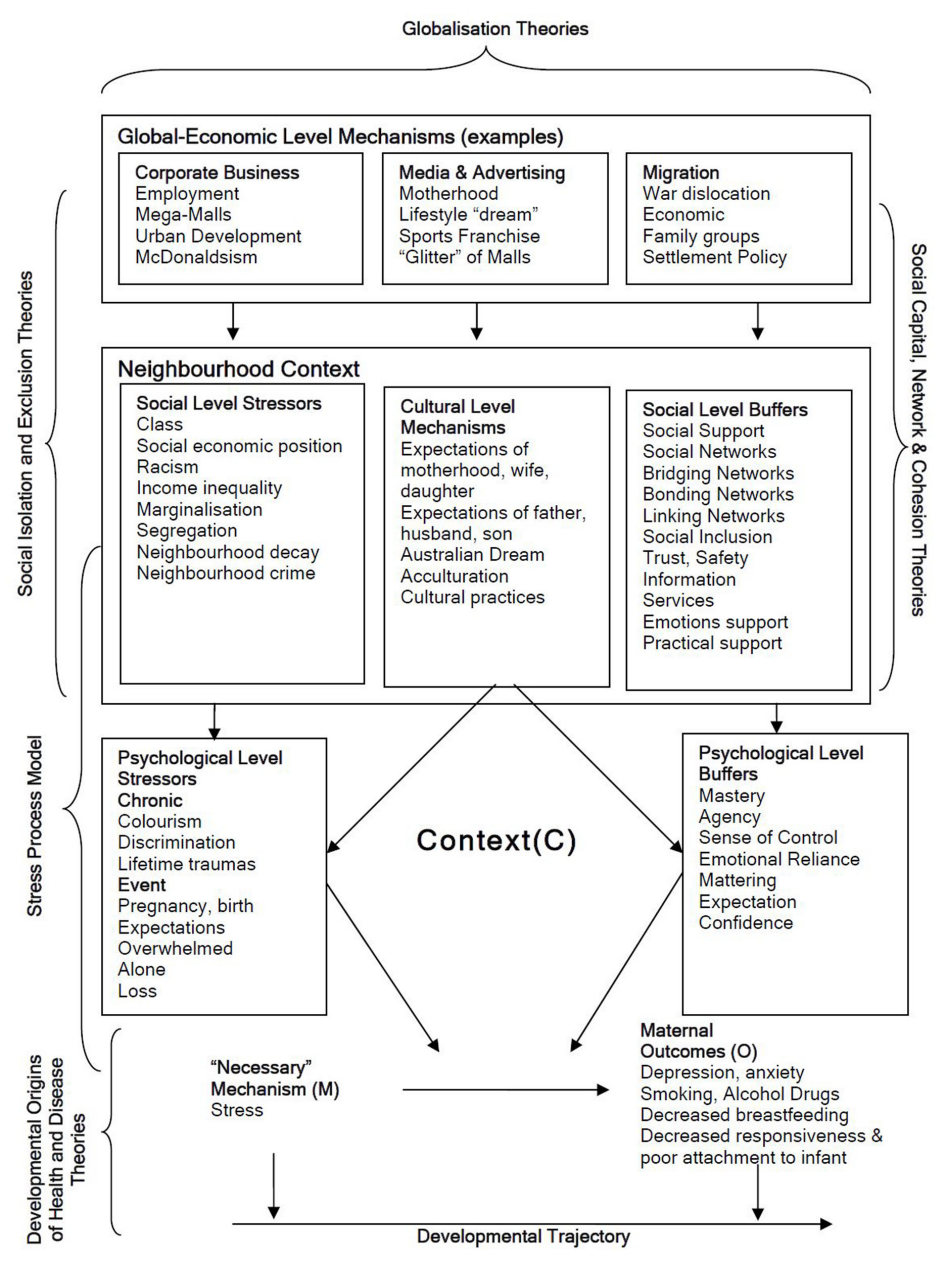
Conceptual Framework of Maternal Depression, Stress and Context [17].

It is well recognised that the early years play an important role in the genesis of later adult health and disease. Current theory construction is focused on how various genetic and environmental mechanisms interact to influence life course outcomes. The environments implicated are: intrauterine, the maternal-infant dyad, family and household life, and external social and physical environments” [16]. Our critical realist theory building analysis [17] used the theoretical frames of: Stress Process; Social Isolation; Social Exclusion; Social Services; Social Capital, Acculturation Theory and Global-economic level mechanisms to explain our observed inequities in maternal outcomes. In our previously reported analysis stress was identified as the underlying “necessary mechanism” that has the tendency to cause several of the observed outcomes including depression, anxiety, and health harming behaviours (Figure 1) [17]. Our ecological and multilevel empirical studies supported the theoretical proposition that neighbourhood adversity causes maternal psychological distress and depression within the context of social buffers including social networks, social cohesion and social services [18]. The theoretical causal propositions from that body of work were subsequently used to generate programme theory which was used in the design study reported here [19,20].

Metropolitan Sydney has been described as being “a city divided” [21,22,23,24,25] with social disadvantage clustered in the southern and western districts. Local perinatal and paediatric social epidemiology studies have further identified socially deprived neighbourhoods and populations with poor perinatal, child and family outcomes [26,27,28,29]. Within Sydney Local Health District (SLHD), located in Central and Inner West Sydney, the clustering of social disadvantage and poor perinatal, child and family outcomes is evident in the Cities of Canterbury, Marrickville and Sydney [28].

The SLHD was established in 2011 and the following year the District commenced a programme of collaborative interagency work to address the needs of children, young people and their families. An early focus of that work was on the special needs of families living with increased psychological and social stress. This paper will describe the development of an intervention design and business case that drew on our earlier realist causal and programme theoretical work and is part of a program of research and programme development that seeks to build and confirm a theory of “Neighbourhood Context, Stress, Depression, and the Developmental Origins of Health and Disease (DOHaD)”. The work was undertaken from 2010 to 2014.

## Theory and Methods

The overall research design is a longitudinal, multilevel, critical realist evaluation of applied programme interventions. The longitudinal dimension involves repeated measures of the output, produced after implementing the program, at different points in the course of the program running (time 1, time 2, time 3). The multilevel aspect incorporates the investigation of different levels of mechanisms operating at the psychological level of self, the level of situated activity, and the levels of intermediate and macro level services. The intervention initiatives, responding to the conceptual framework (Figure 1), were designed and implemented by interagency and community collaborations. In doing this we aimed to move from “explaining underlying social mechanisms to generate social interventions in partnership with the affected populations” [30].

**Figure 2:**
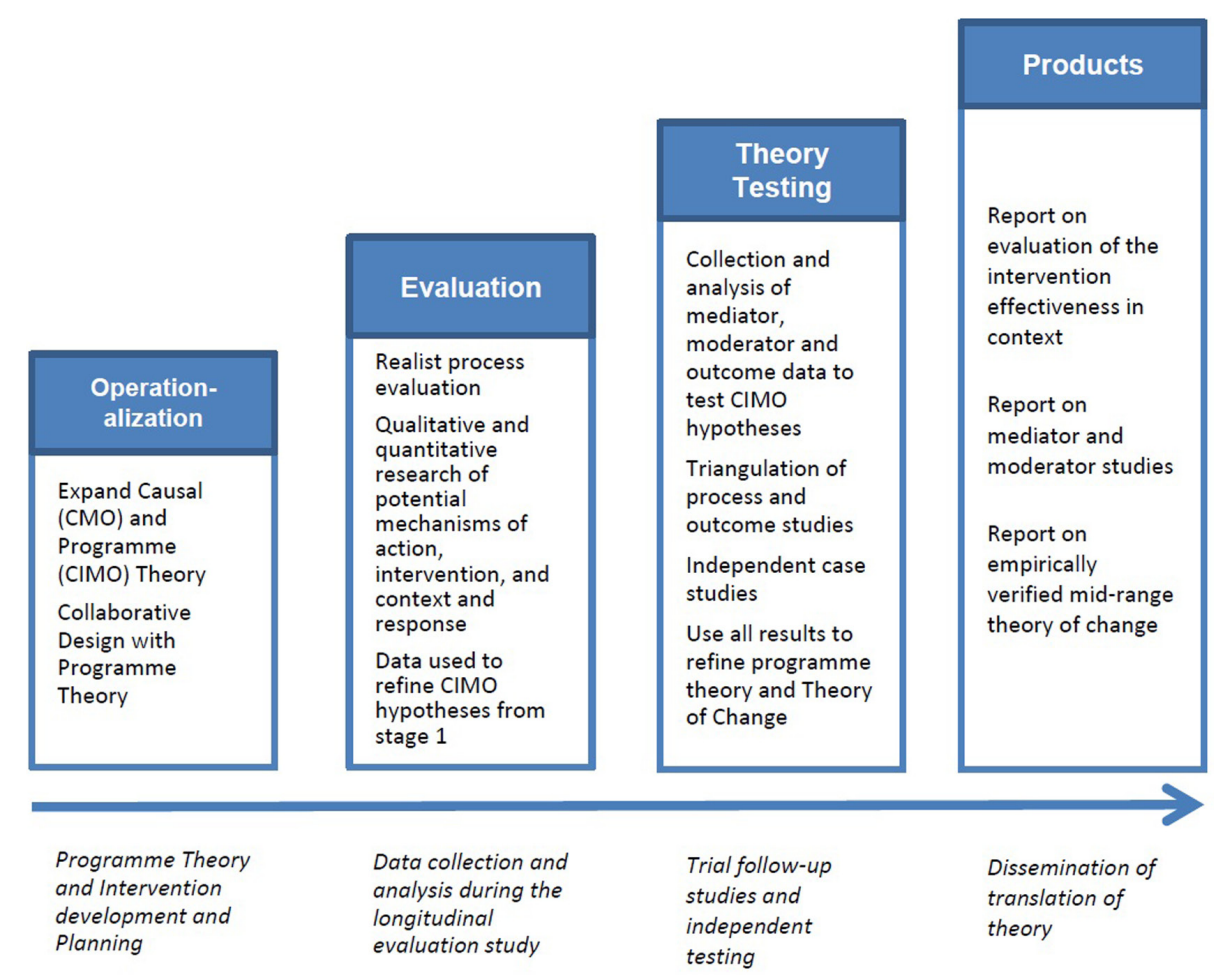
Summary of Research Programme.

The main research programme comprised of four phases (Figure 2). The methodology used for the four phases was reported separately [19]. In summary the four phases are: 1) operationalisation of programme theory and intervention development and planning; 2) evaluation of the interventions; 3) theory testing studies; and 4) dissemination of the findings. In this paper we report on one of the collaborative design projects undertaken in Phase 1: Operationalisation.

### Critical realism and programme design

**Figure 3:**
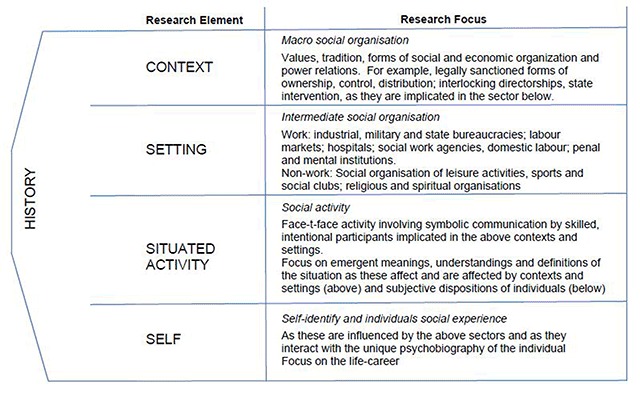
Research Map [31].

Critical realist philosophy of science seeks to discover the underlying mechanisms (M) that cause an empirically observed event or outcome (O). The idea that an event will not always follow from a causal mechanism, in an open system, is called a *tendency*, where the contextual conditions for the mechanisms to operate may not exist. Thus it is important that the nature of the pre-existing conditions be examined. Critical realism also holds that reality is stratified so that each level has its own mechanisms and it is the existence of these level-specific mechanisms that constitute or define a level. The ability of mechanisms to combine to create something new is called *emergence*. Layder [31] illustrated this layering of reality in his Research Map (Figure 3). In this study we will use the following modification of the levels proposed by Layder [31], namely, Self, Situated Activity, Setting -Intermediate Level Social Organisation and Context – Macro Level Social Organisation. Mechanisms, emergence, a hierarchy of levels, and pre-existing historical conditions are all central to the critical realist design process described here.

Realist causal propositions are expressed in terms of mechanisms (M), context (C), and outcomes (O). The MCO propositions in our previously reported theory [17] are in the MCO form proposed by Danermark and colleagues [32]. For evaluation studies, Pawson and Tilley [33] have proposed a CMO configuration. In realist programme evaluation terminology the mechanism (M) is an intervention mechanism (IM), and not a causal mechanism. Denyer and colleagues [34] draw attention to the importance of specifying the intervention separate from the mechanism and proposed the use of a CIMO-logic (Context, Intervention Mechanism, Outcome). Thus a CIMO is a hypothesis that the programme theory produces a change (O) because of the action of an intervention (I) on an underlying mechanism (M) operating in particular contexts (C). We will use the CIMO logic in this study and will apply it to the development of the Theory of Change (ToC) logic model (Figure 6).

Realist programme evaluation usually starts with a programme that has been already designed. The approach assumes that whenever a programme is implemented it is testing an existing programme theory consisting of realist programme hypotheses (CMOs). The process of designing a programme intervention using realist causal and programme theory is not well explicated. For the purposes of this study we have drawn on the work of Keller and colleagues [35] who present a realist design-evaluation framework that combines design theory and realist evaluation.

### Collaborative Design

The collaborative design of the integrated care initiative involved: 1) planning forums; 2) shared outcome planning; 3) collaborative interagency planning; and 4) preparation of a fully funded business plan, Theory of Change and logic model.

The development of a Theory of Change using collective and collaborative processes can be difficult. We used the set of steps proposed by Mackenzie and Blamey [36]:

1. Identification of the long-term outcomes that the initiative seeks to achieve
2. Identification of the interim outcomes and contextual features that will be required to meet these longer-term outcomes
3. Specification of the activities that will be put into place and the contextual requirements to realize these interim outcomes
4. An explicit recognition of the resources that will be required to turn these goals into reality.

The design analysis integrated our earlier causal and programme theoretical work, and collaborative design for vulnerable families. Consequently the critical realist theoretical framework guiding the collaborative effort incorporated 4 elements:

1. A historical analysis of the context to theorise the pre-existing social structures and mechanisms[15]
2. Proposed design elements of an intervention, stemming from inputs from forums, interviews and collaborations during 2013 and 2014
3. The development of a programme theory hypothesising the pre-existing situational conditions and causal mechanisms, and specifying how the proposed intervention would trigger desired psychological, motivational and behavioural responses to bring about change [14]
4. The construction of Theory of Change (ToC) logic model explicating a proposed implementation theory [14].

### Ethics

The planning undertaken here did not include human subjects. Ethical approval was not sought. The indicator reports used secondary data and did not require ethics approval. The earlier cited mixed method multilevel studies had ethics approval from the University of New South Wales.

## Results and Design

### Historical Analysis of the Context

At the level of service providers, the New South Wales (NSW) Government, Australia, introduced an interagency initiative for families in 1999. This was known as *Families First*. The aim of *Families First* was to support families and communities to care for children. The initiative drew on existing services and resources, and had a strong focus on coordinating a network of services. The initiative was later renamed *Families NSW* and has a foundation of local interagency groups supported by programme management groups (PMGs) at District levels. The Inner West Collaborative Programme Management Group (CPMG) plays a significant role in the planning of services for families within SLHD and is a pre-existing social structure with mechanisms that the present design initiative will aim to reconfigure.

At the service to consumers level, in 2009, an epidemiology report of child and family indicators was published that included information on the health and wellbeing of children and families living in both south western and inner west regions of Sydney [27]. In preparation for the design work described here that report was updated in 2013 for Sydney LHD [28]. Secondary analysis from the child and family indicator data-sets was made available for participants of the Vulnerable Children’s Forum and the Supporting Children and Families Forum. That analysis focused in detail on data available for each of the LGAs in SLHD, and was supplemented by a SLHD population needs analysis, and concurrent reviews of perinatal coordination and Infant of Substance Abusing Mothers (ISAM) Pathways [37].

**Figure 4:**
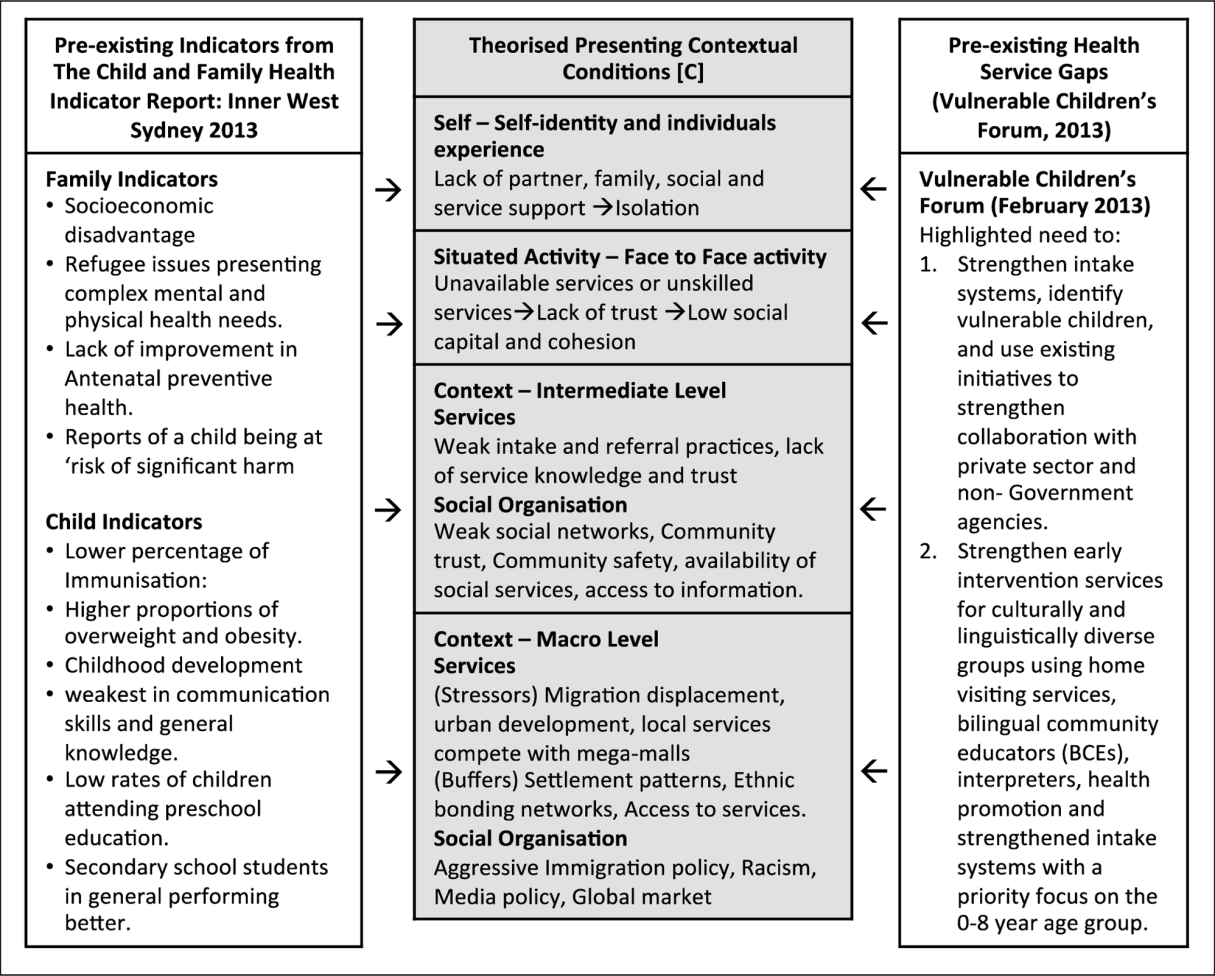
Theorised Contextual conditions.

The updated Child and Family Health Indicator Report: Inner West Sydney 2013 [28] and results from Vulnerable Children’s Forum 2013 highlighted the challenges faced by service consumers in the Inner West Sydney District context and the service gaps that service providers needed to take into account, respectively. Findings from the recently completed study of “Neighbourhood Context, Stress, Depression, and the Developmental Origins of Health and Disease (DOHD)” [17] which elaborated realist causal and program theory were also included. That study was undertaken in the neighbouring South Western Sydney local government areas of Bankstown, Fairfield, Liverpool and Campbelltown. Those information sources, in combination, provided information and supported theories about the presenting contextual conditions [C] as shown in Figure 4 below. The analysis of contextual conditions uses a modification of the four levels proposed by Layder [31].

**Figure 5:**
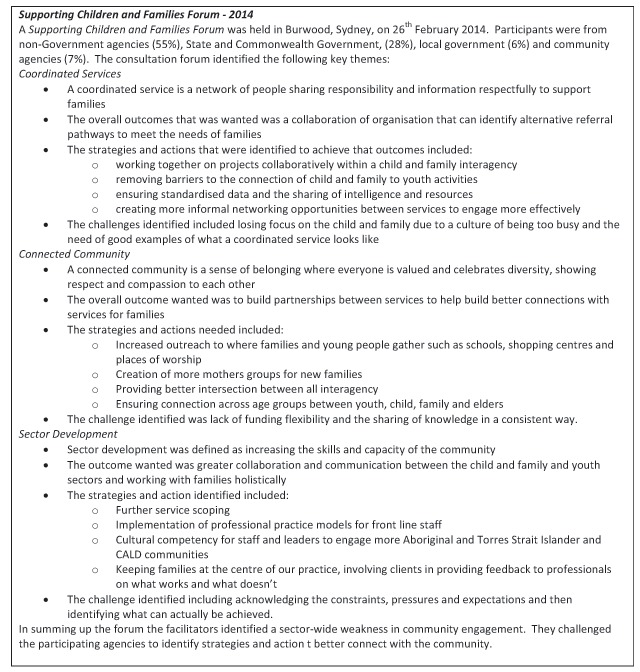
Summary of Consultation Forum.

As earlier mentioned, two consultation forums were held. While the Vulnerable Children’s Forum highlighted service gaps in the context of interest, the Supporting Children and Families Forum 2014, contributed to the collaborative design by identifying the desirable service provision. The planning framework included assessment of: 1) the role of the health sector and SLHD, 2) scientific and economic evidence of program effectiveness; 4) determinants and outcomes of SLHD child health and development; 4) current deployment of SLHD resources; 5) current system performance; and 6) insights from front-line staff and interagency partners. A summary of the outcomes of the 2014 forum is shown below (Figure 5).

### Design Elements

In response to the contextual analyses and consultation forums, a design initiative was formulated which formed the proposed interventions [I].

Three scientifically supported solutions were identified as possible solutions to disrupt the intergenerational cycles of disadvantage observed in SLHD:

1. Sustained Nurse Home Visiting services for vulnerable mothers and their infants until 2 years using a tiered approach [38,39]
2. Intensive “wrap around” counselling models for “high risk” mothers experiencing interpersonal violence, and with complex mental health and substance use problems [40]
3. Preschool and school-based centre and home visiting interventions to reduce conduct disorder, bullying, depression, and alcohol use [40].

Two Programme Logic supported solutions were also identified to support sector wide delivery:

1. Actively managed integration of services and care-coordination (rather than case management) of interventions for high risk infants and their mothers
2. Community-wide and place-based inter-sectoral initiatives that address the social determinants of child and family health [39].

The design elements were developed for inclusion in: 1) a Vulnerable Family Business Case, and 2) Child and Family Health Planning Priorities (Table 1).

**Table 1:** Design Elements.

Design Component	Business Case	Child and Family Health Planning Priorities

Sustained Home Visiting (SHV)	Antenatal screening and risk stratification Perinatal pathways and coordination Sustained home visiting commencing before birth Second tier allied health and medical services, pathways and coordination Universal maternal, child and family services with proportionate support according to need	Review and strengthen perinatal coordination Strengthen Aboriginal SHV (Yana Muru) New SNV in Canterbury LGA focusing on CALD families Enhance SHV in Sydney LGA focusing on Redfern and Waterloo suburbs Strengthen Tier 2 support services including access pathways
Family and Community Integrated Service Development (FCISD)	Integrated service models including wrap-around and family group conference model Targeted parenting programmes Domestic violence intervention High risk infant tracking models “Hub” and “place-based” community building and service coordination Universal family and community capacity building (health and wellbeing promotion)	Interagency collaborative planning Development of interagency models of care for “high need” schools and early childhood centres Commence neighbourhood “hub” development in Redfern social housing estate Enhanced collaborative interagency parenting communication strategy (phone app and web development)
Infrastructure Support (IS)	Child and family public health (epidemiology, programming, research and evaluation) System change strategies Service capacity building Project Management and leadership	Child and family epidemiology Evidence-informed programming Evaluation of perinatal referral pathways Study of universal well child care system Web-based health pathway development Development of well child care and psychological trauma workforce training packages Leadership and technical support to interagency planning groups

*Note*: SHV – Sustained Home Visiting; FCISD – Family and Community Integrated Service Development; IS – Infrastructure Support.

### Programme Theory

The design elements arising from the collaborative design were informed by sound theoretical propositions regarding the underlying programme mechanisms. The programme theory concerns itself with specifying the potential psychological, motivational and behavioural outcomes produced by interventions at each level or layer.

The programme theory in Table 2 below is expressed in realist terms as context-intervention-mechanism and outcome (CIMO) conjectures. The first 2 columns in Table 1 below highlight the pre-existing Contextual Elements [C] and the prevailing causal mechanisms therein [C_M_]. The third to fifth columns highlight the proposed intervention [I], the intervention programme mechanisms [M_P_] and the anticipated outcomes [O] resulting, in response to the intervention. The categorised rows indicate how proposed elements of the intervention will work in configuration with each level of the context identified (Self, Situated Activity, Intermediate Level and Macro Level) using the levels or layers proposed by Layder [31] (Figure 3).

**Table 2:** CIMO Propositions.

Theorised Contextual Conditions (Figure 2)[C]	Present contextual mechanisms activated [C_M_]	Proposed Intervention Design Elements (Table 1)[I]	Postulated Intervention Programme Mechanisms(Table 1)[M_P_]	Postulated psychological, motivational and behavioural Outcomes [O]

Self – Self-identity and individuals experience				

Lack of partner and family support, Distrust of services, Limited treatment access	Stress mechanism activated causing anxiety and depression	Friendship and family support, Professional support, Medication, Treatment	Activate mediating mechanisms of family, peer and professional support to strengthen and build trusting relationships with peers, family and clinicians through SHV and FCISD Design Components.	Decreased depression and anxiety
Lifetime trauma, Loss, Being alone, Isolation	Stress mechanism activated arising from mismatched expectations, and loneliness	Family and peer support, Home visiting, Telephone support		Increased perceived support
**Situated Activity – Face to Face activity**				

Services unavailable or poor access, Services not trusted, Services not skilled	Absence of trusted professional support mechanism	“wrap around” services, Family Conferences, Workforce training	Activate services mechanisms that are client, peer and neighbourhood focused, and trauma and evidence informed through FCISD and IS Design Components.	Improved perceived access to skilled and trusted services
Community distrust, Low social capital and cohesion, crime, unemployment	Absence of trusted neighbourhood and community support mechanism	“wrap around” services, Family Conferences, Public health, Social work services		Improved perceived support from neighbours and community
**Intermediate Level social and service organisation**				

Unhelpful intake and referral practices, Lack of service, knowledge and trust	Absence of specialist service support mechanism for front-line professionals	Strengthened pathways and design Collocation of services	Activate mechanisms related to trust and confidence with service network, increased local social capital, community trust and community safety Activate mechanisms relating to improved coordination and access to services and information through FCISD and IS Design Components.	Improved perceived access to services that are “wrapped” around front-line workers
Weak social networks, community trust, community safety, available social services, access to information	Social level stress mechanisms relating to class, position, racism, segregation, crime and neighbourhood decay are activated tending to increase psychological stress	Population and community level interventions in neighbourhoods and communities		Decrease in psychological stress of individuals and families
**Macro Level social and service organisation**				

Migration, Mega-malls pull service activity away from neighbourhoods, Urban development	Activation of social level stress mechanisms tend to hinder the activation of social level buffer mechanisms	Population and community level interventions in neighbourhoods and communities	Activate mechanisms related to increased social level activities in deprived neighbourhoods. Activate mechanisms related to increased migrant related social activities among ethnic populations through FCISD and IS Design Components.	Increase in perceived social level buffers
Immigration policy, Racism, Media policy, Global market, Settlement patterns, Ethnic bonding networks, Access to services	Migrant related social level mechanisms including acculturation, cultural practices and integration tend to decrease social level stress	Ethnic and cultural specific community and population level interventions		Increase in perceived migrant social level buffers

### Theory of Change (ToC)

**Figure 6:**
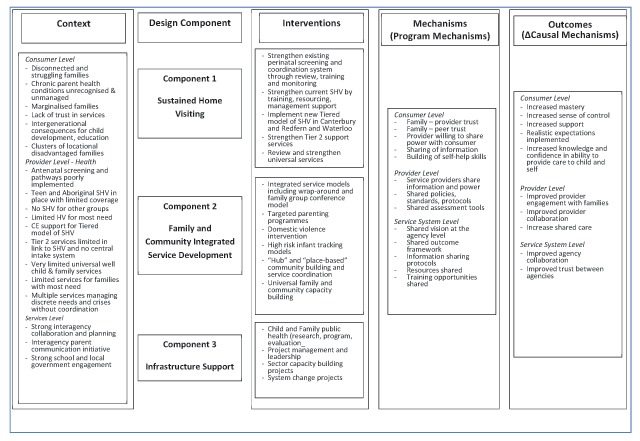
ToC Logic Model.

The ToC Logic Model (Figure 6) outlines the hypothesised links between the underlying programme mechanisms (programme theory), the intervention activities (implementation theory), and how they are anticipated to work in synergy to bring about desired outcomes [14].

The ToC Logic model was constructed following steps recommended by Mackenzie and Blamey [36]. Those steps are:

1. Identification of the long-term outcomes that the initiative seeks to achieve
2. Identification of the interim outcomes and contextual features that will be required to meet these longer-term outcomes
3. Specification of the activities that will be put into place and the contextual requirements to realize these interim outcomes
4. An explicit recognition of the resources that will be required to turn these goals into reality [36].

## Discussion

We have used critical realist meta-theory to assist in the translation of previously reported empirical explanatory theory building to theory driven interventions. In so doing we have aimed to move from identifying and explaining the underlying social and psychological causal mechanisms, toward generating evidence-informed social interventions in partnership with the affected populations.

We demonstrate the design of interventions for vulnerable families in Sydney utilising translational research from previous realist causal and program theory building to operational service design. For example, previously developed propositions about the underlying mechanisms that cause maternal stress (i.e. loneliness and lack of trust) were used to develop propositions regarding programme mechanisms (i.e. trusting relationships) and design elements (i.e. sustained home visiting) that might buffer this effect.

Local quantitative and qualitative studies were used together with consultation forums and collaborative design approaches. Central to the development of the collaborative design reported here was the infrastructure provided by a network of interagency collaborative groups established as part of the earlier Families NSW initiative. Both consultative forums contributed to the development of shared long-term and interim outcomes and the identification of activities that if put in place could realise those outcomes. Resources necessary to initiate those activities were identified and included into a health sector business plan.

### Limitations

The critical realist approach requires the inclusion of an analysis of pre-existing structures and mechanisms that may be contributing to the observed maternal, child and family outcomes [15]. The use of research findings from neighbouring South Western Sydney introduced a weakness into the design process which was only partly offset by the local forum, stakeholder interviews and the perinatal drug health study [37]. A further limitation of the analysis and design elements was the strong health sector focus despite the collective approach to planning. This weakness was partly attributable to significant restructuring of the NSW Departments of Education, and Family and Community Services, which was undertaken during the planning process.

The programme theory used to inform the intervention remains tentative and will require testing during the implementation phase. The design propositions developed followed the context-intervention-mechanism-outcome (CIMO) logic proposed by Denyer [34]. We are not aware of this approaching being applied previously to the translation of causal theory to programme theory. We propose that the robustness of this approach be assessed as part of the evaluation of the design implementation.

## Conclusion

In undertaking this study we identified the importance of our earlier analysis of underlying causal mechanisms and related programme mechanisms for identifying the elements for the full intervention design. The application of theory added rigour to the design of the integrated care initiatives. In applying the theory to the local situation the analysis took into account: the role of the local agencies; evidence of program effectiveness; determinants and outcomes for local children and their families; the current deployment of service resources; and insights from front-line staff and interagency partners.

## Abbreviations

CPMG: Collaborative Programme Management Group, DOHaD: Developmental Origins of Health and Disease, CMO: Context, Mechanism, Outcome, CIMO: Context, Intervention, Mechanism, Outcome, ISAM: Infant of Substance Using Mother, NSW: New South Wales, SHV: Sustained Home Visiting, SLHD: Sydney Local Health District, ToC: Theory of Change, TDE: Theory Driven Evaluation.

## References

[1] Australian Institute of Health and Welfare. A picture of Australia’s Children 2012 Canberra: AIHW; 2012.

[2] Nicholson, JM, Lucas, N, Berthelsen, D and Wake, M. Socioeconomic inequality profiles in physical and developmental health from 0–7 years: Australian National Study. J Epidemiol Community Health, 2012; 66(1): 81–7. DOI: 10.1136/jech.2009.103291 20961874

[3] Durey, A and Thompson, SC. Reducing the health disparities of Indigenous Australians: Time to change focus. BMC Health Serv Res, 2012; 12: 151–62. DOI: 10.1186/1472-6963-12-151 22682494 PMC3431273

[4] Baum, F and Fischer, M. Are the national preventive health initiatives likely to reduce health inequalities. Aust J Prim Health, 2011; 17(4): 320–6. DOI: 10.1071/PY11041 22112700

[5] Gluckman, PD and Hanson, MA. (eds.) The Developmental Origins of Health and Disease Cambridge: Cambridge University Press; 2006 DOI: 10.1017/CBO9780511544699

[6] Kuh, D and Ben-Shlomo, Y. A life course approach to chronic disease epidemiology Oxford: Oxford University Press; 1997. 11980781

[7] Thornberry, TP and Henry, KL. Intergenerational continuity in maltreatment. J Abnorm Child Psychol, 2013; 41(4): 555–69. DOI: 10.1007/s10802-012-9697-5 23192742 PMC3640695

[8] Najman, JM, Aird, R, Bor, W, O’Callaghan, M, Williams, GM and Shuttlewood, GJ. The generational transmission of socioeconomic inequalities in child cognitive development and emotional health. Soc Sci Med, 2004; 58(6): 1147–58. DOI: 10.1016/S0277-9536(03)00286-7 14723909

[9] Thornberry, TP, Knight, KE and Lovegrove, PJ. Does maltreatment beget maltreatment? A systematic review of the intergenerational literature. Trauma, Violence, & Abuse, 2012; 13(3): 135–52. DOI: 10.1177/1524838012447697 PMC4035025 22673145

[10] Woodruff, J and O’Brien, J. Children’s and family services working together. Australian Journal of Early Childhood, 2005; 30(1): 49–7. DOI: 10.1177/183693910503000109

[11] Wood, J. Report of the Special Commission of Inquiry into Child Protection Servicse in NSW. State of NSW through the Special Commission of Inquiry into Child Protection; 2008.

[12] Council of Australia Governments. Protecting children is everyone’s business: National framework for protecting Australia’s children 2009–2020 In: Governments CoA, (ed.). Canberra ACT: Commonwealth of Australia; 2009.

[13] Muntaner, C. Invited commentary: on the future of social epidemiology—a case for scientific realism. Am J Epidemiol, 2013; 178(6): 852–57. DOI: 10.1093/aje/kwt143 24008904

[14] Blamey, A and Mackenzie, M. Theories of change and realistic evaluation peas in a pod or apples and oranges? Evaluation, 2007; 13(4): 439–55. DOI: 10.1177/1356389007082129

[15] De Souza, DE. Elaborating the Context-Mechanism-Outcome configuration (CMOc) in realist evaluation: A critical realist perspective. Evaluation, 2013; 19(2): 141–54. DOI: 10.1177/1356389013485194

[16] Eastwood, J, Kemp, B and Jalaludin, B. Each is in Different Circumstances Anyway: A Realist Multilevel Situational Analysis of Maternal Depression in South Western Sydney, Australia. SAGE Open, 2016: 1–14. DOI: 10.1177/2158244016676863

[17] Eastwood, JG, Kemp, BA and Jalaludin, BB. Realist theory construction for a mixed method multilevel study of neighbourhood context and postnatal depression. SpringerPlus, 2016; 5: 1081 DOI: 10.1186/s40064-016-2729-9 27468381 PMC4945545

[18] Eastwood, JG, Jalaludin, BB, Kemp, LA and Phung, HN. Neighbourhood adversity, ethnic diversity, and weak social cohesion and social networks predict high rates of maternal depressive symptoms: A critical realist ecological study in South Western Sydney, Australia. Int J Health Serv, 2013; 43(2): 241–66. DOI: 10.2190/HS.43.2.d 23821904

[19] Eastwood, J, Kemp, L, Garg, P, Tyler, I and De Douza, D. A Critical Realist Translational Social Epidemiology Protocol for Concretising and Contextualising “Theory of Neighbourhood Context, Stress, Depression, and the Developmental Origins of Health and Disease (DOHaD)”, Sydney Australia. Int J Integr Care, 2019; 19(3): 8, 1–13. DOI: 10.5334/ijic.3962 PMC6659581 31367207

[20] Eastwood, J. Building Realist Program Theory for Interventions for Vulnerable Children and Families in Sydney, Australia. Int J Integr Care, 2017; 17(5). DOI: 10.5334/ijic.3539

[21] Flood, J. Sydney divided: factorial ecology revisited. Residential differentiation in Australian; 2000.

[22] Waitt, G. Social impacts of the Sydney Olympics. Annals of Tourism Research, 2003; 30(1): 194–215. DOI: 10.1016/S0160-7383(02)00050-6

[23] Birrell, B and Healy, E. Metropolis divided: the political dynamic of spatial inequality and migrant settlement in Sydney. People and Place, 2003; 11(2): 65.

[24] Forrest, J, Lean, G and Dunn, K. Challenging racism through schools: teacher attitudes to cultural diversity and multicultural education in Sydney, Australia. Race Ethnicity and Education, 2015: 1–21. DOI: 10.1080/13613324.2015.1095170

[25] Vinson, T, Rawsthorne, M, Beavis, A and Ericson, M. Dropping off the edge 2015: persistent communal disadvantage in Australia Canberra, Australia: Jesuit Social Services & Catholic Social Services Australia; 2015.

[26] Morgan, K, Eastwood, J and Faniran, S. Headline population indicators data report 2009: A report for the South West Sydney region on key statistics relating to the health and wellbeing of its children and families Liverpool, NSW: Karitane & Sydney South West Area Health Service; 2009.

[27] Fisher, K, Brown, M, Halim, S, Hendry, A and Eastwood, J. Child and family health indicators report: South Western Sydney 2013 Liverpool, NSW: South Western Sydney Local Health District; 2013.

[28] Alexander, K, Brown, M, Halim, S, Hendry, A and Eastwood, J. Child and Family Health Indicators Report: Inner West Sydney 2013 Croydon, NSW, Australia: Sydney Local Health District; 2015.

[29] Chong, S, Nelson, M, Byun, R, Harris, L, Eastwood, J and Jalaludin, B. Geospatial analyses to identify clusters of adverse antenatal factors for targeted interventions. Int J Health Geogr, 2013; 12: 46 DOI: 10.1186/1476-072X-12-46 24152599 PMC4016259

[30] Muntaner, C. Invited Commentary: Social Mechanisms, Race, and Social Epidemiology. Am J Epidemiol, 1999; 150(2): 121–6. DOI: 10.1093/oxfordjournals.aje.a009970 10412956

[31] Layder, D. New Strategies in Social Research: An Introduction and Guide Cambridge, UK: Polity Press; 1993.

[32] Danermark, B, Ekstrom, M, Jakobsen, L and Karlsson, J. Explaining Society: Critical realism in the social sciences London: Routledge; 2002.

[33] Pawson, R and Tiley, N. Realistic Evaluation London: Sage; 1997.

[34] Denyer, D, Tranfield, D and Van Aken, JE. Developing design propositions through research synthesis. Organization studies, 2008; 29(3): 393–413. DOI: 10.1177/0170840607088020

[35] Keller, C, Gare, K, Edenius, M and Lindblad, S. Innovations in health care: Design theory and realist evaluation combined. Innovations. 2010; 11: 22–2010.

[36] Mackenzie, M and Blamey, A. The Practice and the Theory: Lessons from the Application of a Theories of Change Aproach. Evaluation, 2005; 11(2): 151–68. DOI: 10.1177/1356389005055538

[37] Alexander, J, Raman, S, Yoong, T and Mawhinney, B. Improving Pathways to Assessment and Care for Infants of Substance Abusing Mothers: Are We Getting It Right? Social Sciences, 2015; 4(1): 192–204. DOI: 10.3390/socsci4010192

[38] Kemp, L, Harris, E, McMahon, CA, Matthey, S, Vimpani, G, Anderson, T, et al. Maternal Early Childhood Sustained Home-visiting (MESCH) Program Manual 2nd ed. Sydney: Centre for Health Equity Training Research and Evaluation (CHETRE), part of the Center for Primary Health Care and Equity, Faculty of Medicine, University of NSW; 2012.

[39] Tolan, P and Dodge, K. Children’s Mental health as a Primary Care and Concern. Am Psychol, 2005; 60(6): 601–14. DOI: 10.1037/0003-066X.60.6.601 16173893 PMC2745240

[40] Center on the Developing Child at Harvard University. A Science-Based Framework for Early Childhood Policy: Using Evidence to Improve Outcomes in Learning, Behaviour, and Health for Vulnerable Children: http://www.developingchild.harvard.edu; 2007.

